# STLV-1 Commonly Targets Neurons in the Brain of Asymptomatic Non-Human Primates

**DOI:** 10.1128/mbio.03526-22

**Published:** 2023-02-21

**Authors:** Brenda Rocamonde, Sandrine Alais, Rodolphe Pelissier, Valerie Moulin, Brigitte Rimbaud, Romain Lacoste, Noemie Aurine, Camille Baquerre, Bertrand Pain, Yuetsu Tanaka, Cyrille Mathieu, Hélène Dutartre

**Affiliations:** a Centre International de Recherche en Infectiologie, équipe d’Oncogenèse Rétrovirale, INSERM U1111 - Université Claude Bernard Lyon 1, CNRS, UMR5308, Ecole Normale Supérieure de Lyon, Université Lyon, Lyon, France; b Centre International de Recherche en Infectiologie, équipe Immunobiologie des Infections Virales, INSERM U1111 - Université Claude Bernard Lyon 1, CNRS, UMR5308, Ecole Normale Supérieure de Lyon, Université Lyon, Lyon, France; c Station de Primatologie-UAR846-CNRS, France; d Univ Lyon, Université Lyon 1, INSERM, INRAE, Stem Cell and Brain Research Institute, U1208, USC1361, Bron, France; e Department of Immunology, Graduate School of Medicine, University of the Ryukyus, Nishiharacho, Okinawa, Japan; f Centre International de Recherche en Infectiologie équipe Neuro-Invasion, TROpism and VIRal Encephalitis, INSERM U1111 - Université Claude Bernard Lyon 1, CNRS, UMR5308, Ecole Normale Supérieure de Lyon, Université Lyon, Lyon, France; g Equipe labellisée par la Fondation pour la Recherche Médicale, Labex Ecofect; National Institutes of Health; Johns Hopkins Bloomberg School of Public Health

**Keywords:** HTLV-1, neurotropism, inflammation, microglial response

## Abstract

The human T-cell leukemia virus (HTLV)-1 is responsible for an aggressive neurodegenerative disease (HAM/TSP) and multiple neurological alterations. The capacity of HTLV-1 to infect central nervous system (CNS) resident cells, together with the neuroimmune-driven response, has not been well-established. Here, we combined the use of human induced pluripotent stem cells (hiPSC) and of naturally STLV-1-infected nonhuman primates (NHP) as models with which to investigate HTLV-1 neurotropism. Hence, neuronal cells obtained after hiPSC differentiation in neural polycultures were the main cell population infected by HTLV-1. Further, we report the infection of neurons with STLV-1 in spinal cord regions as well as in brain cortical and cerebellar sections of postmortem NHP. Additionally, reactive microglial cells were found in infected areas, suggesting an immune antiviral response. These results emphasize the need to develop new efficient models by which to understand HTLV-1 neuroinfection and suggest an alternative mechanism that leads to HAM/TSP.

## OPINION/HYPOTHESIS

The human T-cell leukemia virus (HLTV)-1 affects 10 to 20 million people worldwide. After a long asymptomatic phase (20 to 30 years), between 1 and 5% of HTLV-1-infected subjects will develop a neurodegenerative disease known as HTLV-1-associated myelopathy/tropical spastic paraparesis (HAM/TSP) (1). HAM/TSP is manifested as an ensemble of motor dysfunctions that evolve toward the paralysis of lower limbs, a consequence of an immune-mediated demyelinated thoracic cord (2). Corticoid therapy is unable to stop the progression of the disease, and no effective treatment has been developed due to the poor comprehension of the mechanisms initiating demyelination. A sustained, exacerbated inflammation triggered by infiltrated lymphocytes has been proposed as the main mechanism (3). Inflammatory and HTLV-1-specific CD8^+^ T-cell lymphocytes, both infiltrated and clonally expanded, can be found in the spinal cord sections (4) and Cerebrospinal Fluid (CSF) of HAM/TSP postmortem samples (5), suggesting a CSF-compartmentalized antigen-driven immune response (presumably HTLV-1 specific) at later stages of the disease. *In vitro* experiments have shown the susceptibility of microglia, astrocytes, and neurons (originated from both tumoral cell lines and primary cells) to HTLV-1 infection (6–9), and HTLV-1 RNA was found in spinal cord astrocytes via the *in situ* hybridization of the postmortem tissues of HAM/TSP patients (10). However, the *in vivo* infection of other neuronal populations remains unclear (11), and it is not fully understood whether their infection is sufficient to initiate T-cell-dependent chronic inflammation. Notably, investigating HTLV-1 neurotropism is a challenging task due to (i) the absence of suitable *in vitro* models that can recapitulate the complexity of the Central Nervous System (CNS) cytoarchitecture, (ii) the limited access to myelinated postmortem samples from infected individuals, and (iii) the lack of tools that are available to be used to investigate HTLV-1 infections (i.e., GFP-reportable viral particles), compared to infections by other retroviruses. In contrast, naturally STLV-1-infected nonhuman primates (NHP) represent a valuable animal model for HLTV-1 infection, as the simian homolog of HTLV-1 shares more than 99% of its genomic homology (12) and offers the possibility of accessing neural tissue before the demyelinating phase of the infection.

To get insights into HTLV-1 neurotropism, we first infected human induced pluripotent stem cells (hiPSC)-derived neural cell polycultures with an HTLV-1-infected cell line via co-cultivation. Then, we analyzed CNS samples from a cohort of nonhuman primates that were naturally infected by STLV-1 to investigate HTLV-1 neural infection *in vivo*. This unique model allowed us to investigate HTLV-1 neurotropism during the latent phase of the disease, specifically, before the manifestation of neural inflammation. Hence, we demonstrated STLV-1 neurotropism *in vitro* and detected, for the first time, the presence of viral proteins in neurons from the spinal cord and cortical regions of STLV-1-infected NHP. Strikingly, few microglia and astrocytes were positive for viral proteins. In contrast, we found reactive microglia in close apposition to infected neurons, suggesting a local immune response to STLV-1 infection that is potentially responsible for an early inflammatory response to the infection. Taken together, these findings raise new hypotheses on the mechanisms that trigger the neural inflammation that was observed in HAM/TSP.

## RESULTS

### HTLV-1 preferentially infects neuronal cells in human iPSC-derived neural polycultures.

**FIG 1:**
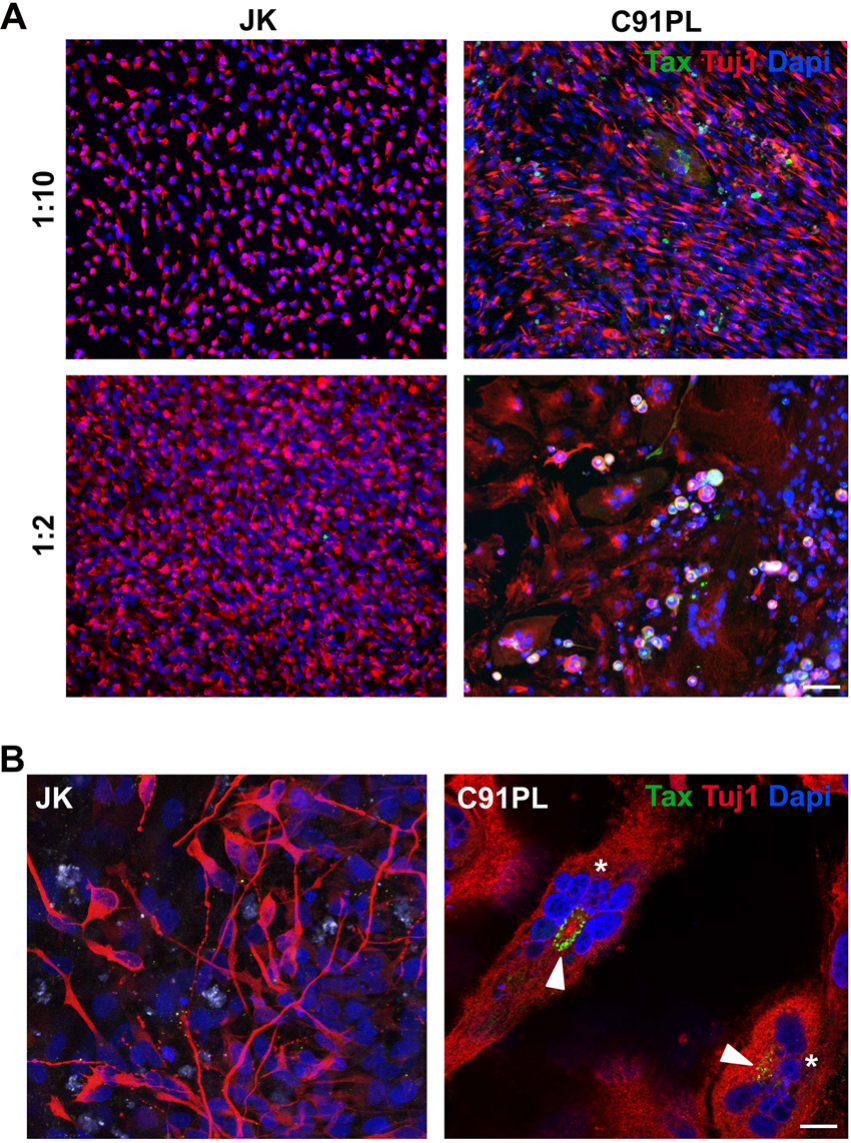
HTLV-1 infects neural cells and triggers a glial response. (A) hiPSC-derived neural cell polyculture cocultured with either JK or C91PL cells at two different ratios for five days. Scale bar: 100 μm. (B) Tax^+^ cells (arrowheads) in hiPSC presenting different morphologies, and polynucleated cells (asterisks) expressing the Tuj1 neuronal marker. Scale bar: 20 μm.

We used human iPSC-derived neural polycultures to investigate neuronal susceptibility to HTLV-1 infection *in vitro*. The productive infection of neural cells co-cultured with C91-PL cells was monitored by the expression of the HTLV-1 Tax oncoprotein, as previously reported (13). Tax expression was detected mainly in neuronal cells expressing beta-III-Tubulin (Tuj1) (Fig. 1A), suggesting the productive infection of the neuronal cells. In these cells, Tax was mainly located in the perinuclear cytoplasm (Fig. 1B, arrowhead). Infection was also associated with the modification of the cell morphology, especially with the highest ratio of infected cells, with polynucleated cells and/or cells presenting fragmented nuclei suggesting a cytopathic effect (Fig. 1B, asterisks).

### NHP were naturally infected with STLV-1 to model HTLV-1 neuroinfection *in vivo*.

**FIG 2:**
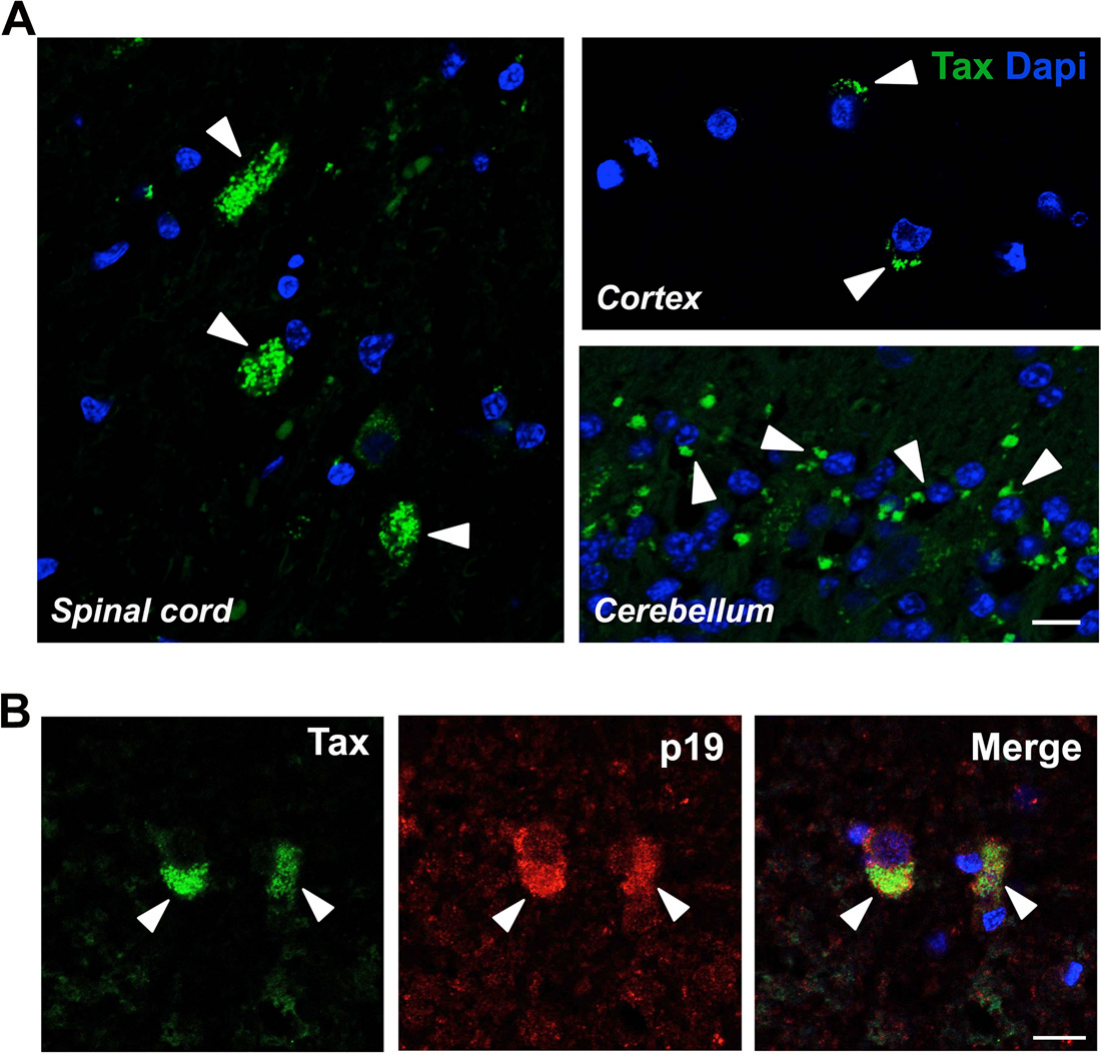
Detection of the HTLV-1 viral proteins in the CNS of naturally SLTV-1-infected NHP. (A) Sections from the spinal cord, cortex, and cerebellum of naturally STLV-1-infected NHP expressing the Tax protein (arrowheads). Scale bar: 50 μm. (B) Colocalization of the Tax and Gag p19 proteins of STLV-1 in spinal cord sections of NHP. Scale bar: 20 μm.

Next, we addressed the question of neuronal susceptibility to HTLV-1 infection *in vivo* due to its extremely high similarity to STLV-1. We took advantage of a cohort of naturally STLV-1-infected NHP to investigate the viral neurodistribution. In this cohort, the average age of NHP at the moment of the analysis was 23 years, with an unknown time of latent infection. No motor dysfunction was reported by the animal keepers during the routine observation of the animals, although no specific motor test was performed to address motor functionality. An overall low proviral load (PVL) was measured in blood periferal blood mononuclear cells (PBMC) (0.06 to 630 copies/10^5^ cells) (Table 1). Strikingly, immunohistochemistry in the spinal cord, cerebellum, and cortex revealed the presence of the viral Tax protein in almost all of the animals (Fig. 2A; Table 1). The frequency of Tax^+^ cells in the spinal cord-infected regions were between 10% and 25% of the total cells analyzed, and no correlation was identified with the PVL. The Tax protein was predominantly localized in the perinuclear cytoplasm, which is consistent with our *in vitro* observations and with previous reports of Tax expression in *in vitro* infected primary cells or cell lines (8). Additionally, we detected the concomitant cytoplasmic expression of the Gag p19 matrix protein of STLV-1 in the Tax^+^ cells (Fig. 2B), suggesting the productive infection of the neural cells.

**TABLE 1:** List of naturally SLTV-1-infected and naive NHP, together with their PVL, and the presence of Tax^+^ cells in the spinal cord, cortex, and cerebellum^*a*^

Number	STLV-1	PVL (copies/10^5^ PBMC)	% cells Tax^+^ spinal cord	Tax^+^	
				Cerebellum	Cortex
01	Neg	na	na	na	na
02	Neg	na	na	na	na
03	Pos	55	23.35	+	+
04	Pos	0.06	19.44	+	+
05	Pos	395	15.12	+	+
06	Pos	68	9.99	+	+
07	Pos	54	18.72	+	+
08	Pos	nd	14.14	+	+
09	Pos	630	17.50	+	+
10	Pos	112	0	−	−

a na, not assessed; nd, not determined.

### STLV-1 in the CNS of naturally infected NHP confirms neuronal tropism.

**FIG 3:**
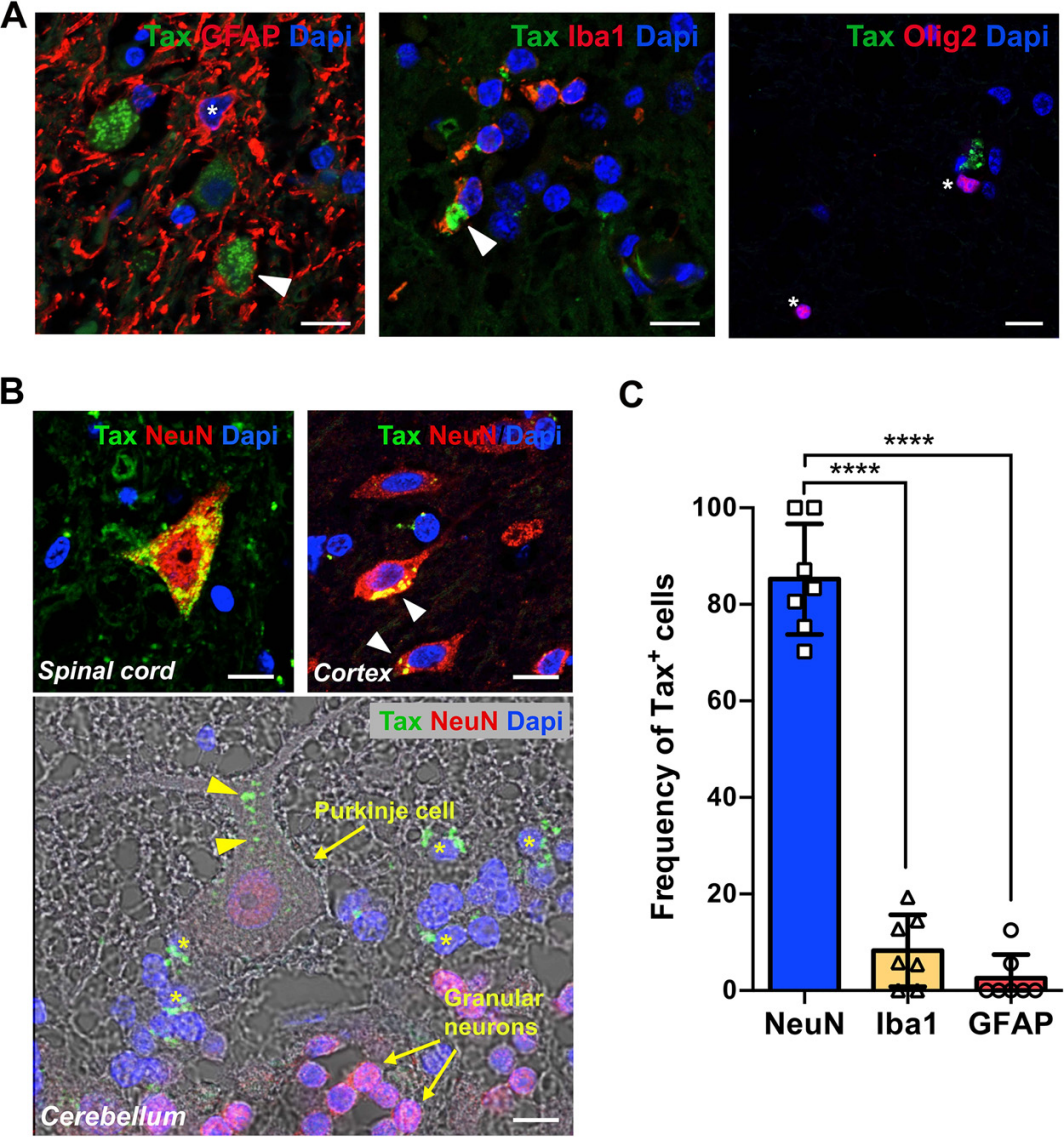
Infection of neural cell populations in naturally STLV-1-infected NHP. (A) Expression of Tax protein (arrowheads) in astrocytes (GFAP^+^, asterisks), microglial cells (Iba1^+^), and oligodendrocytes (Olig2^+^, asterisks). Scale bar: 50 μm. (B) Tax protein expression in NeuN^+^ neurons from the spinal cord, cortex, and cerebellum. Scale bar: 20 μm. (C) Bar plot representing the frequency of Tax^+^ cells in the NeuN^+^, GFAP^+^, and Iba1^+^ cells from spinal cord sections of NHP.

HTLV-1 DNA was previously reported in the astrocytes of postmortem spinal cord samples from a HAM/TSP patient (10). However, the HTLV-1 infection of other neural cell types was not investigated or reported. Thus, we addressed whether other CNS resident cell types could be infected by STLV-1 in naturally infected NHP. As expected, the Tax protein was detected in 2% of the spinal cord astrocytes, and it was identified based on their expression of GFAP, confirming their susceptibility to the infection, as in human cases (Fig. 3A). Unexpectedly, microglial cells were also positive for Tax staining (10% of Iba1^+^ cells), whereas the oligodendrocyte lineage cells expressing the transcription factor Olig2 were Tax negative (Fig. 3A). Consistent with our previous *in vitro* observations, the Tax protein was mainly localized in NeuN^+^ neurons in the spinal cord as well as in the cortical regions (Fig. 3B, up). In the cerebellum, staining was localized in the ganglionic layer but not in NeuN^+^ neurons. A few big neuronal cells from the Purkinje cells/Golgi cells layer also expressed Tax (Fig. 3B, down). Such results confirm that STLV-1 can reach at least the cortical and cerebellar regions. Overall, the frequency of Tax in the CNS resident cells showed that almost 80% were NeuN^+^ neurons, 20% were Iba1^+^ microglial cells, and few were GFAP^+^ astrocytes (Fig. 3C), indicating a specific neuronal tropism of STLV-1.

### STLV-1 is associated with a local neuroimmune response.

**FIG 4:**
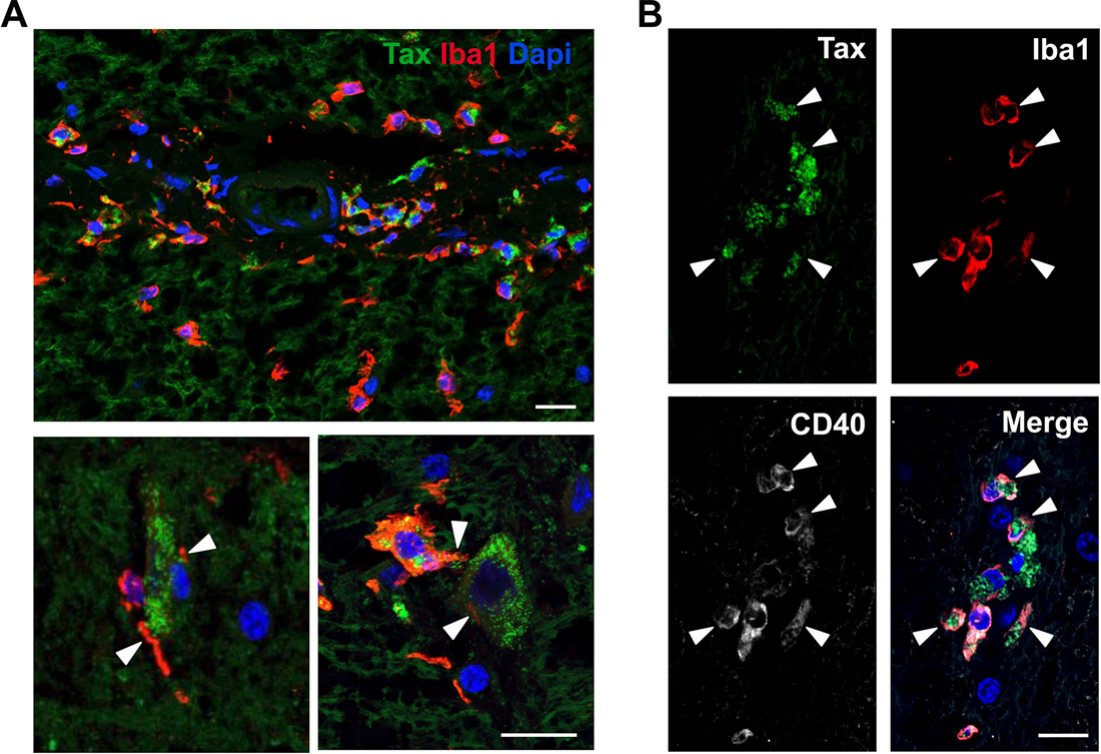
Neuroimmune response to HTLV-1 infection. (A) Microglia cells (Iba1^+^) localized nearby Tax^+^ cells in spinal cord sections of NHP. Arrowheads point to extended processes contacting infected cells. Scale bar: 50 μm. (B) Microglial cells (Iba1^+^) expressing the activation marker CD40 Scale bar: 50 μm. A one-way ANOVA that was followed by a Fisher’s post-test was performed to determine statistical significance. ****, *P* < 0.0001.

Numerous microglial cells (Iba1^+^) that were positive or not for the infection were frequently localized in areas surrounding infected cells (Fig. 4A). Notably, in several spinal cord regions, microglial cells were found in close apposition to Tax^+^ cells (most likely neuronal cells). Some of these surrounding microglial cells showed an amoeboid morphology, with extended processes that contact Tax^+^ cells (Fig. 4A, arrowheads). This microglial cell phenotype is characteristic of activated phagocytic microglia. Moreover, the expression of the CD40 marker (Fig. 4B, arrowheads), a reliable indicator of microglia activation, confirmed the presence of reactive microglia in the infected areas.

## DISCUSSION

To date, the mechanisms involving HTLV-1-driven neurodegeneration are still not well-understood. The number of neurological affections triggered by HTLV-1 infection is probably underestimated, and information regarding the cell-type specific neurotropism is limited. Previous studies have reported the *in vitro* infection of astrocytes, microglia, and neuronal monocultures (6–8). However, the frequency of infected cells and the differential susceptibility among cell types due to the bystander effect of neighboring cells have not been evaluated. Here, we showed that neurons are more susceptible to HTLV-1 infection than glial cells on both hiPSC-derived *in vitro* neural polycultures and postmortem samples from NHP. Interestingly, the expression of the HTLV-1 Tax protein in our *in vitro*-infected neurons was associated with polynucleated cells. Morphological and nuclear alterations on HTLV-1-infected cells have been previously reported. Indeed, Tax was reported to induce genetic instability and increase the frequency of mutations, thereby inducing important morphological and functional changes that resulted in micro/multinuclei development (14–16). The impact of Tax-induced chromatin modifications on neuronal survival and function remains to be addressed. Cognitive dysfunction in some HTLV-1-infected individuals (17) might be reminiscent to neuronal infection. Most neural HTLV-1 manifestations are observed in HAM/TSP patients who are infected during adulthood. However, the HTLV-1 neuroinfection of newborns during mother-to-child transmission (18) could lead to more severe defects, as neural progenitors are more susceptible to cell cycle deregulations or induced apoptosis. Several cases of HAM/TSP were reported in adolescents who were breastfed by mothers infected with HTLV-1 (19), suggesting that the impact of HTLV-1 infection on neural development may be underestimated.

The *in vivo* assessment of HTLV-1 neurotropism remains a challenging task due to the complexity of accessing CNS samples from HTLV-1-infected individuals. The *in situ* hybridization of spinal cord postmortem tissue from HAM/TSP patients revealed the presence of HTLV-1 DNA in astrocytes from the spinal cord (10). However, the infection of other resident cells of the CNS was not reported. The analysis of demyelinated samples may neglect the susceptibility of different cellular populations that are sensitive to cell death or tissue atrophy. The analysis of postmortem CNS samples from naturally infected NHP allowed us to detect the presence of the HTLV-1 Tax and Gag p19 proteins in different neuronal subtypes *in vivo*, suggesting the productive infection of neurons. While several neuronal subpopulations were found to be positive for STLV-1 infection, such as pyramidal and motor neurons, granular neurons remained Tax negative. A differential tropism among neuronal populations was suggested to occur with other viruses, such as Vesicular Stomatitis Virus (VSV), in which serotonin and norepinephrine neurons were selectively infected (20). The mechanisms driving the selective STLV-1 neurotropism remain unknown, but they could respond to (i) opportunity (access to a certain region), (ii) the absence of restriction factors that allow for cell infection in susceptible neurons, and/or (iii) the differential expression of viral receptors that allow for cell entry. Neuropilin-1, one of the known viral receptors of HTLV-1, is highly expressed by endothelial cells and neurons (21), and it could be utilized to facilitate HTLV-1 infection. Indeed, the infection of endothelial cells seems to be a plausible route of entry into the CNS (22, 23).

The high frequency of infected cells in the spinal cord of asymptomatic NHP, together with the infection of the cortical and cerebellar regions, suggests a more extensive viral spread within the CNS than it was previously reported. Magnetic resonance imaging (MRI) has revealed lesions in several CNS areas, such as cortical white matter regions, even before symptomatic manifestations (24), suggesting that demyelination is not restricted to the spinal cord. Activated microglial cells were found in close apposition of Tax^+^ cells in infected areas and may reflect the sensing and induction of an immune response to the infection. The engulfment of infected neurons could trigger inflammation within the CNS, together with their clearance in later stages. Multiple and quite diverse neuropathies, other than HAM/TSP typical symptomatology, have been reported in HTLV-1 seropositive subjects, such as acute disseminated encephalomyelitis, meningitis, myopathies, or peripheral neuropathies (25, 26), the development of which are still not well-understood. These pieces of evidence lead us to hypothesize an important role of microglia in neuroinflammation and interrogate whether its response to neuronal infection could be at the origin of HTLV-1-associated neuropathies and at the onset of HAM/TSP. In conclusion, our results showed that the impact of HTLV-1 neuroinfection might be underestimated, as HAM/TSP manifestation is only the late stage of a progressive, aggressive, and complex immunological response to neuroinfection. Furthermore, our study emphasizes the use of naturally STLV-1-infected NHP as a relevant model with which to investigate STLV-1 invading and spreading within the CNS. This approach would pave the way for the identification of biomarkers of neuroinflammation before HAM/TSP symptomatic manifestations.

## MATERIALS AND METHODS

### Ethics statement.

The use of animals was approved by the Ethics Committee No. 14 (APAFIS no. 4227–201604130940121) of the French Minister of Education and Research. Animals were housed at the primate center of the CNRS (UAR 846) in Rousset-sur-Arc and were cared for in compliance with French regulations. The experimental procedure complied with the current French laws and the European directive 86/609/CEE. Blood was obtained after anesthesia via the intramuscular injection of ketamine (5 mg/kg) and medetomidine (0.05 mg/kg), and CNS samples were obtained after euthanasia via the intravenous injection of pentobarbital (180 mg/kg).

### Animals.

Eight naturally STLV-1-infected baboons (Papio anubis) were included in this study. Two animals were STLV-1 naive. HTLV-1 serology was performed to discriminate HTLV-1-positive animals from HTLV-1-negative animals. Then a polymerase chain reaction (PCR) was performed on the positive animals to determine the proviral load (PVL). All of the analyzed animals were females, and the average age at the moment of the euthanasia was 23 years.

### Proviral load measurement.

The PVL was measured from isolated PBMC. Briefly, PBMC were isolated from total blood via Ficoll gradient after centrifugation. After two washes in phosphate-buffered saline (PBS), the cells were centrifuged. Genomic DNA was extracted from the cell pellets using a NucleoSpin Blood Kit (Macherey-Nagel, Düren, Germany). The DNA concentration was determined using a NanoDrop ND-1000 spectrophotometer (Thermo Scientific).

Real-time PCR was performed with 20 to 40 ng of genomic DNA for controls as well as 100 to 120 ng for virus amplification, using the FastStart Universal SYBR Green Master (Roche, reference number 4913850001), in 50 μL of the final volume. The primer sequences for the viral amplification were selected in the *tax* gene: F-5′-GTTGTATGAGTGATTGGCGGGGTAA and R-5′-TGTTTGGAGACTGTGTACAAGGCG. The primer sequences for control amplification were selected on the β-actin gene (27). The STLV-1 copy numbers were determined as the number of copies for 1 × 10^5^ cells. The sensitivity was determined to be 10 copies of the Tax amplicon.

### CNS sample processing and immunofluorescence.

The brain and the first segment of the spinal cord were harvested after the euthanasia of the animals. The spinal cord, motor cortex, and cerebellum were dissected and fixed in 4% paraformaldehyde (PFA) for 48 h. The samples were embedded in paraffin wax (Sigma-Aldrich, catalog number P3558) and sectioned at 5 μm. The slides were dewaxed in xylene (VWR; 3 times for 5 min) and rehydrated in successive baths of EtOH 95%, 70%, and 30%. After 5 min in water, the slides were incubated with a sodium citrate solution (10 mM sodium citrate, Sigma; 0.05% Tween 20, VWR; pH = 6) in a boiling water bath for 20 min for heat-induced epitope retrieval and were washed in PBS. The samples were then incubated with blocking buffer (PBS, 3% BSA, 0.15% Triton X-100) for 20 min. Then, the sections were stained in blocking buffer overnight at 4°C with mouse anti-Tax (1:100, courtesy of Y. Tanaka) (28), mouse anti-Gag p19 (1:100, Zeptometrix), rabbit anti-GFAP (1:500, Dako), guinea pig anti-NeuN (1:500, Sigma), goat anti-Iba1 (1:200, Wako), polyclonal goat anti-hOlig2 (1:100, catalog number AF2418, R&D Systems) or rabbit anti-CD40 (1:100, Ozyme).

After 3 washes in PBS for 5 min, the slides were incubated with anti-mouse Alexa Fluor 488, anti-goat Alexa Fluor 555, anti-guinea pig Alexa Fluor 555, and anti-rabbit Alexa Fluor 647 conjugated antibodies as well as with DAPI (Invitrogen) that was diluted in blocking buffer (1:750, Life Science Technologies). Finally, the sections were mounted on glass slides with Fluoromount G (SouthernBiotech) before observation via confocal microscopy (Zeiss LSM800) using 40× and 63× oil objectives.

For the quantifications, triplicate sections of each animal were analyzed. Between three to five images were analyzed in which positive cells were identified. The number of positive cells was divided by the total number of cells per field. A one-way ANOVA followed by a Fisher’s post-test was performed to determine statistically significant differences in the numbers of Tax^+^ cells between neural cell populations.

### Human iPSC-derived neural polycultures.

Human embryonic fibroblasts (HEF), kindly provided by Odile Boespflug-Tanguy (AP-HP, Robert Debre Hospital, Department of Neuropediatrics and Metabolic Diseases, National Reference Center for Leukodystrophies, Paris, France), were reprogrammed into hiPSCs using a CytoTune Sendai Reprogramming Kit (Life Technologies), according to the manufacturer’s instructions. Once isolated, the clones were amplified, fully characterized, registered in the Human Pluripotent Stem Cell Registry (https://hpscreg.eu/) and declared through the Codecoh DC-2020-3895. Dissociated hiPSC cells were plated at 1.5 × 10^5^ cells in a poly-l-ornithine/laminin-coated well containing a neural induction medium (NIM) that was composed of DMEM/F-12 complemented with 2 mM l-glutamine, 1,000 U/mL penicillin-streptomycin, 1% MEM nonessential amino acids solution, 1 mM 2-mercaptoethanol and 1% N-2 supplement. The medium was changed every 2 days, and the cells were changed into neural stem medium that was supplemented with 20 ng/mL of human recombinant basic fibroblast growth factor (hrFGF; 154 AA, Peprotech) and 20 ng/mL of murine recombinant epidermal growth factor (mrEGF; Peprotech) after 7 days. After 14 days, the medium was supplemented with 0.5 μM ATRA, 2% B-27 supplement, and 100 ng/mL of human recombinant Sonic Hedgehog (hrSHH, StemCell Technologies). At day 28, the NIM was complemented with 2% B-27 supplement, 100 ng/mL of hrSHH, and 10 ng/mL of hrFGF for 2 days. The cells were then used for viral testing after 30 days.

### HTLV-1-infected cell lines and cocultures.

Polycultures derived from hiPSCs were differentiated and matured for 30 days to ensure the presence of neurons, astrocytes, and oligodendrocytes. The obtained polycultures were cocultured with the HTLV-1-chronically infected lymphocyte cell line C91-PL for 5 days at 2 different cell ratios (1:10 and 1:2). C91-PL cells were previously incubated with mitomycin (50 μg/mL, Sigma) for 20 min to stop the progression of the cell cycle. After 23 h, the cultures were flushed with PBS, and the medium was changed every other day. On the fifth day, the cells were fixed with 4% PFA and rinsed with PBS. Staining using anti-Tax (1:100) and anti-Tuj1 (1:500) antibodies was performed directly in the ibidi 24-well plates (BioValley). Images were taken using an inverted microscope Zeiss Axio Observer Z1 with a confocal unit LSM 980, and the images were analyzed using the ImageJ software 1.52p Fiji package.
